# Secreted Frizzled Related Protein 5 (SFRP5) Serum Levels Are Decreased in Critical Illness and Sepsis and Are Associated with Short-Term Mortality

**DOI:** 10.3390/biomedicines11020313

**Published:** 2023-01-22

**Authors:** Philipp Hohlstein, Jonathan F. Brozat, Julia Schuler, Samira Abu Jhaisha, Maike R. Pollmanns, Lukas Bündgens, Theresa H. Wirtz, Eray Yagmur, Karim Hamesch, Ralf Weiskirchen, Frank Tacke, Christian Trautwein, Alexander Koch

**Affiliations:** 1 Department for Gastroenterology, Metabolic Disorders and Intensive Care Medicine, University Hospital RWTH Aachen, Pauwelsstraße 30, 52074 Aachen, Germany; 2 Institute of Laboratory Medicine, Western Palatinate Hospital, 67655 Kaiserslautern, Germany; 3 Institute of Molecular Pathobiochemistry, Experimental Gene Therapy and Clinical Chemistry (IFMPEGKC), University Hospital RWTH Aachen, Pauwelsstraße 30, 52074 Aachen, Germany; 4 Department of Hepatology and Gastroenterology, Charité–Universitätsmedizin Berlin, Campus Virchow-Klinikum (CVK) and Campus Charité Mitte (CCM), Augustenburger Platz 1, 13353 Berlin, Germany

**Keywords:** intensive care unit, critical illness, sepsis, human, cytokine, adipokine, biomarker, inflammation, immune system, organ failure, prognosis, CARS

## Abstract

Sepsis is a major health burden with insufficiently understood mechanisms of inflammation and immune paralysis, leading to a life-threatening critical illness. The secreted frizzled related protein 5 (SFRP5) acts as an anti-inflammatory adipokine by antagonizing the Wnt5a pathway. The aim of this study was to elucidate the role of SFRP5 in critical illness and sepsis and to determine its value as a prognostic biomarker for mortality. We analyzed SFRP5 serum concentrations of 223 critically ill patients at admission to a medical intensive care unit (ICU) and compared those to 24 healthy individuals. SFRP5 serum concentrations were significantly decreased in critical illness as compared to healthy controls (24.66 vs. 100 ng/mL, *p* = 0.029). Even lower serum concentrations were found in septic as compared to nonseptic critically ill patients (19.21 vs. 32.83 ng/mL, *p* = 0.031). SFRP5 concentrations correlated with liver disease, age, anti-inflammation, and metabolic parameters. Furthermore, patients with sepsis recovered levels of SFRP5 in the first week of ICU treatment. SFRP5 levels at admission predicted short-term mortality in critically ill but not in septic patients. This study points to the role of the anti-inflammatory mediator SFRP5 not only in sepsis but also in nonseptic critically ill patients and associates high levels of SFRP5 to worse outcomes, predominantly in nonseptic critically ill patients.

## 1. Introduction

Sepsis and septic shock remain major health care problems, affecting millions of patients and leading to death in around one third of those patients [1]. Major efforts have been made in improving timely antibiotic treatment and supportive care of sepsis; however, the exact mechanisms leading to sepsis remain unclear [2]. Septic patients show an initially uncontrolled pro-inflammatory response, formerly known and described as the systemic inflammatory response syndrome (SIRS), which includes an anti-inflammatory response to regulate counter-responses [3]. In fact, this inappropriate systemic host response, leading to life-threatening organ dysfunction, constitutes the most recent definition of sepsis [4]. However, the comprehension of the process behind this uncontrolled pro-inflammatory response and its determination remains to be elucidated. Defects of regulatory pathways in the innate as well as in the adaptive immunity have been described [5,6,7]. Furthermore, this anti-inflammatory state can be prolonged, leading to a compensatory anti-inflammatory response syndrome (CARS), which might cause excess morbidity and mortality [3,8,9].

The wingless-type mouse mammary tumor virus (MMTV) integration site family member 5a (Wnt5a) is expressed in cells of adipose tissue, macrophages and CD14 positive monocytes [10] and plays a regulatory role in proinflammatory pathways [11,12,13,14,15]. Wnt5a polarizes the differentiation away from M1-type macrophages and towards M2-type macrophages [15] and increases endothelial permeability, leading to increased cell migration and angiogenesis [12]. The secreted frizzled-related proteins (SFRPs) are part of this cytokine system with a family of five known secreted glycoproteins [16,17]. Those proteins contain a netrin-like functional domain and a cysteine-rich domain, both exhibiting a close homology with the frizzled cysteine-rich domain contained in the Wnt/β-catenin signaling pathway, which causes accumulation of β-catenin in the affected cell, ultimately leading to reading of Wnt target genes [18,19,20]. SFRP5 is an antagonist of this pathway and was first described in human retinal pigment epithelial cells [21]. SFRP5 is produced and released from adipose tissue, therefore playing a role as an anti-inflammatory adipokine [22,23,24,25,26,27,28]. By blocking this pathway, SFRP5 suppresses chronic inflammation, attenuates insulin sensitivity, and increases lipid accumulation in adipocytes as well as their differentiation [16,29,30]. Alteration and regulation of SFRP5 serum levels have been found in type 2 diabetes, obesity, and non-alcoholic steatohepatitis [16,18,22,31,32,33,34]. Furthermore, SFRP5 has shown favorable effects on atherosclerotic cardiovascular disease and was increased in chronic heart failure [35,36].

Given the close links between metabolism and inflammation seen in the Wnt pathway, one might suspect that SFRP5 also plays a role in the dysregulated immune response in critical illness and sepsis. In fact, in a small study with 60 critically ill patients, the authors showed a dysregulation of the Wnt5a/SFRP5 system in human sepsis but did not observe statistical alterations in SFRP5 concentrations [37]. In another research letter, higher concentrations of Wnt5a were demonstrated in patients with acute respiratory distress syndrome due to SARS-CoV-2 [38]. Aside from those studies, the role of SFRP5 in human sepsis remains unclear. Therefore, we conducted a large clinical study focusing on the association between SFRP5 and organ dysfunction, regulation of SFRP5 in critical illness and sepsis as well as possible function as a prognostic biomarker.

## 2. Materials and Methods

### 2.1. Study Design

This study was conducted as a retrospective, observational cohort study to investigate the role of SFRP5 in critically ill patients treated in a medical intensive care unit. Written informed consent was obtained from the patient, his or her spouse, or the appointed legal guardian. We enrolled 223 patients admitted to our medical intensive care unit of the Department of Gastroenterology, Digestive Disease, and Intensive Care Medicine. We included patients with consent, age above or equal to 18 and available blood samples on the day of ICU admission as previously described [39,40]. Patients (1) with expected short-term (<48 h) intensive care treatment, (2) patients admitted from another ICU, and (3) patients admitted due to poisoning were excluded. To retrospectively discriminate between septic and nonseptic patients, the Third International Consensus Definition for Sepsis was applied (4). Follow-up data was collected by contacting the patient, his/her relatives, or the primary care physician.

As a control group we recruited healthy blood donors (n = 24) from the local blood bank with normal values for blood counts and liver enzymes as well as absent chronic disease. In addition, ongoing infection or acute or chronic disease were excluded by a clinical examination. This study was approved by the local ethics committee (EK150/06) of the University Hospital Aachen, RWTH Aachen and was conducted in agreement with the 1964 Declaration of Helsinki.

### 2.2. SFRP5 Measurements

We collected blood samples at the time of admission to the intensive care unit (day 1) and one week after ICU admission, if applicable. Blood samples were centrifuged at 4 °C for 10 min and serum aliquots of 1 mL were frozen immediately at −80 °C until further use. SFRP5 concentrations were measured using a commercially available ELISA according to the instructions of the manufacturer (Wuhan USCN Business Co., Ltd., USCNK, No. 33 ZhenHua Road, Wuhan Economic & Technological Development Zone, Wuhan, China). SFRP5 measurements were performed blinded to any clinical or other laboratory data of patients or controls.

### 2.3. Statistical Analysis

Data were analyzed using SPSS Version 29 (SPSS, Chicago, IL, USA). Data were given as median and range due to the skewed distribution of most parameters. As normal distribution of samples could not be assumed, the two-tailed Mann–Whitney U test or chi-squared test were used for two groups of unpaired samples, and the two-tailed Wilcoxon signed-rank test was used for paired samples. A significance level of *p* = 0.05 was used in all corresponding calculations. Patient survival was depicted by Kaplan–Meier curves followed by a log rank-, Breslow-, and Tarone-Ware-test for level of significance. The Youden index (the sum of sensitivity and specificity minus one) was calculated to identify optimal cut-off values for parameters to discriminate prognosis. The correlation between parameters was assessed by Spearman’s rank correlation test.

## 3. Results

### 3.1. SFRP5 Levels Are Decreased in Critical Illness and Sepsis

The study cohort consisted of 147 patients admitted due to sepsis and septic shock and 76 patients admitted due to nonseptic critical illness. Median age was 63 years with a range of 18 to 90 years and did not differ between septic and nonseptic patients. There were also no differences in sex, comorbidities (assessed using the Charlson Comorbidity Index), need of mechanical ventilation, or short-term mortality for death in the ICU or over a 30-day period between the study cohorts of septic and nonseptic patients. However, patients with sepsis showed higher APACHE II and SOFA scores, had more demand for vasopressors and needed longer treatment in the intensive care unit. Furthermore, septic patients had a higher 1-year mortality as compared to nonseptic patients (Table 1).

Most septic patients were treated due to pulmonary sepsis (n = 83). Other infection sites were the abdomen (n = 22) or the urogenital tract (n = 10). Furthermore 32 septic patients were treated for other infection sites such as blood stream infections, skin infections or an unknown focus. Nonseptic patients were admitted due to cardiopulmonary disorders (n = 17), respiratory failure without infection (n = 12), advanced liver disease (n = 17), or various other diseases (n = 30) (Table 2).

**Figure 1:**
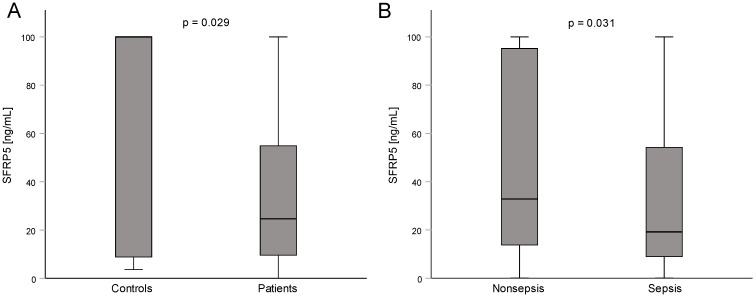
Concentrations of SFRP5 in healthy controls with a median of 100 ng/mL (**A**) and in critically ill patients with and without sepsis (**B**). For comparison of two groups, the Mann–Whitney U test was used. Sample sizes: controls n = 24, patients n = 223, nonsepsis n = 76, sepsis n = 147.

To examine the potential regulation of circulating SFRP in critically ill patients, we compared serum SFRP5 levels at admission to the intensive care unit to healthy control samples. In healthy controls, median SFRP5 concentration was 100 ng/mL (which was the upper limit of the measurement range) while ICU patients showed significantly lower serum concentrations of SFRP5 than healthy controls, with a median of 24.66 ng/mL (*p* = 0.029, Figure 1A and Table 1). Additionally, septic patients had significantly lower levels of SFRP5 when compared to nonseptic patients (19.21 vs. 32.83 ng/mL, *p* = 0.031, Figure 1B and Table 1).

### 3.2. SFRP5 Levels Are Dependent of Age and Comorbidities, but Independent of Sex and BMI in Critical Illness

**Figure 2:**
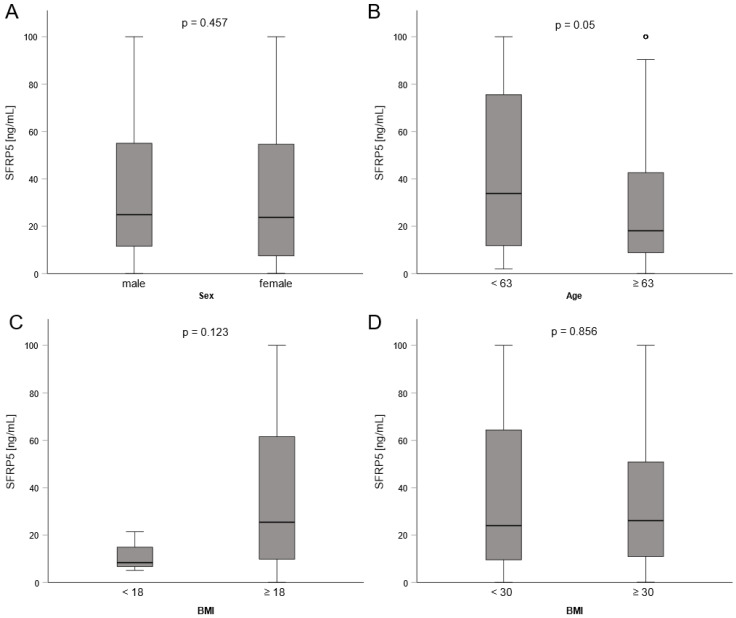
Levels of SFRP5 of critically ill patients treated on the ICU and their comparison between gender (**A**), Age in years (**B**) and body-mass index (BMI) in kg/m2 (**C**,**D**). For comparison of two groups, the Mann–Whitney U test was used. Sample sizes: patients n = 223.

Next, we aimed at evaluating whether SFRP5 serum concentrations differ between demographic subgroups. We observed no significant differences between male and female patients (Figure 2A). Concerning the body-mass index (BMI) we did not observe alterations in SFRP5 levels between groups of patients above or below a BMI of 18, nor 30 kg/m^2^ (Figure 2, Table 3). Interestingly, older patients (above or equal to the median age of 63) had lower levels of SFRP5 (Figure 2B). This was also reflected in a modest negative correlation between age and SFRP5 (Spearman’s r = −0.138, *p* = 0.040, Table 3).

To evaluate other influence factors of SFRP5 serum concentrations, we reviewed comorbidities of intensive care patients. Notably, presence of diabetes, arterial hypertension, and chronic obstructive pulmonary disease had no influence on serum levels of SFRP5. Nevertheless, patients with known liver disease as well as chronic alcohol abuse showed higher serum levels of SFRP5, but this only reached statistical significance for patients with liver disease (*p* = 0.005). Patients with coronary artery disease as well as patients with active malignancy showed a tendency towards lower levels of SFRP5, again without reaching significance (*p* = 0.063 and *p* = 0.056, respectively, Table 4).

### 3.3. SFRP5 Levels Correlate with Biomarkers of (Anti-)Inflammation as well as Hepatic and Lipid Metabolism

To further investigate other potential stimuli of SFRP5 serum concentrations we performed extensive correlation analyses between SFRP5 levels and demographical and clinical characteristics, as well as a broad selection of laboratory measurements for different organ functions such as renal, liver, coagulator function and markers of inflammation. Furthermore, days in the intensive care unit also had a modest negative correlation to SFRP5 levels (Spearman’s r = −0.155, *p* = 0.021). Disease severity and organ failure (SOFA and APACHE II scores) on day one or three showed no correlation to admission levels of SFRP5 (Table 3).

Regarding markers of inflammation status, we observed a weak positive correlation to blood leukocytes (Spearman’s r = 0.133, *p* = 0.048) and hemoglobin levels (Spearman’s r = 0.140, *p* = 0.036). Furthermore, we detected a weak negative correlation of procalcitonin and SFRP5 (Spearman’s r = −0.175, *p* = 0.028) and a moderate negative correlation of SFRP5 serum concentration and Interleukin 10 (Spearman’s r = −0.302, *p* < 0.001). Moreover, C-reactive protein and Interleukin 6 did not correlate with SFRP5 levels. In contrast, we did not observe any correlations to electrolytes, functional markers of the renal system nor functional/clinical markers of the cardiopulmonary system. Interestingly, for markers of the hepato-pancreatico-biliary and coagulation system we observed weak to moderate correlations of SFRP5 levels to total serum protein levels (Spearman’s r = 0.238, *p* < 0.001), activated partial thromboplastin time (aPTT, Spearman’s r = −0.154, *p* = 0.023) and lipase (Spearman’s r = 0.147, *p* = 0.049). Interestingly, markers of liver function and inflammation (INR, bilirubin, Gamma-GT, AST and ALT) did not correlate with SFRP5 levels. For markers of the metabolic and endocrine system we observed positive weak to moderate correlations of SFRP5 concentrations to total cholesterol (Spearman’s r = 0.173, *p* = 0.019), the high-density lipoprotein (HDL, Spearman’s r = 0.201, *p* = 0.046), and the low-density lipoprotein (LDL, Spearman’s r = 0.265, *p* = 0.008). Glucose and insulin levels did not correlate to SFRP5 concentrations (Table 3). To evaluate the influence of age on all other correlations to SFRP5 levels, we additionally conducted partial correlations controlled for age. Here, almost all correlations were retained except for lipase levels *(p* = 0.066 in partial correlation).

### 3.4. Septic Patients Recover Levels of SFRP5 after One Week of Intensive Care Treatment

**Figure 3:**
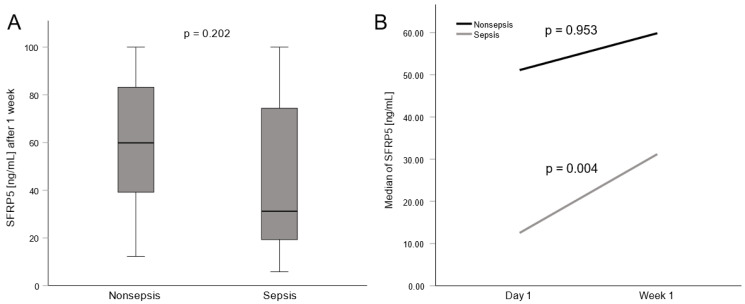
SFRP5 concentrations and change thereof in critically ill patients on the ICU. (**A**) SFRP5 concentrations after one week of treatment on the ICU in septic and nonseptic patients. (**B**) Change of SFRP5 concentrations after one week of treatment on the ICU in septic and nonseptic patients. For comparison of two unpaired groups, the Mann–Whitney U test, and for comparison of two paired groups the Wilcoxon signed rank test was used. Sample sizes: nonsepsis n = 10, sepsis n = 32.

Additional serum samples after one week of ICU treatment were available in 42 patients of the cohort. We analyzed those serum samples to gain understanding of SFRP5 trends in the course of the critical disease. Considering the lower levels of SFRP5 in septic patients compared to nonseptic patients on admission day, we related those groups again after one week and could not find any significant differences (Figure 3A). To underline the trend of SFRP5 concentrations in individual patients we compared levels of SFRP5 at admission to levels after one week. Here, we observed an increase in circulating SFRP5 in most septic patients (Wilcoxon z = 2.841, *p* = 0.004). In contrast, we found equally high values of SFRP5 levels in nonseptic patients (Wilcoxon z = −0.059, *p* = 0.953; Figure 3B).

### 3.5. SFRP5 Levels at Admission to the ICU Are Predictors of Short-Term Mortality in Critical Illness but Not Sepsis

**Figure 4:**
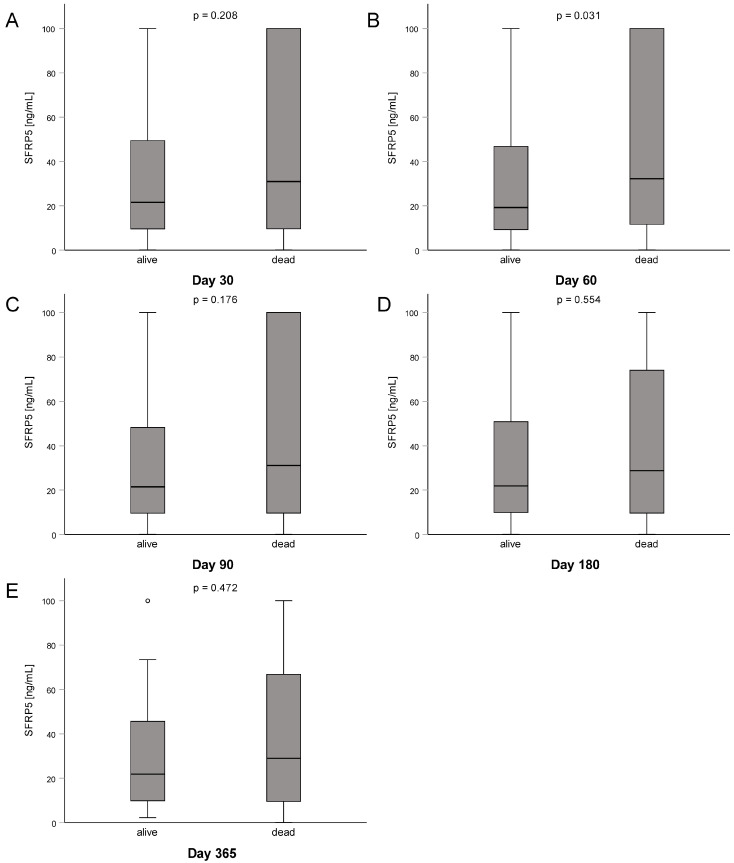
Consecutive survival analysis of critically ill patients treated on the ICU. (**A**–**E**) Survival status on days 30 through 365. For comparison of two groups, the Mann–Whitney U test was used. Sample sizes: patients n = 223.

**Figure 5:**
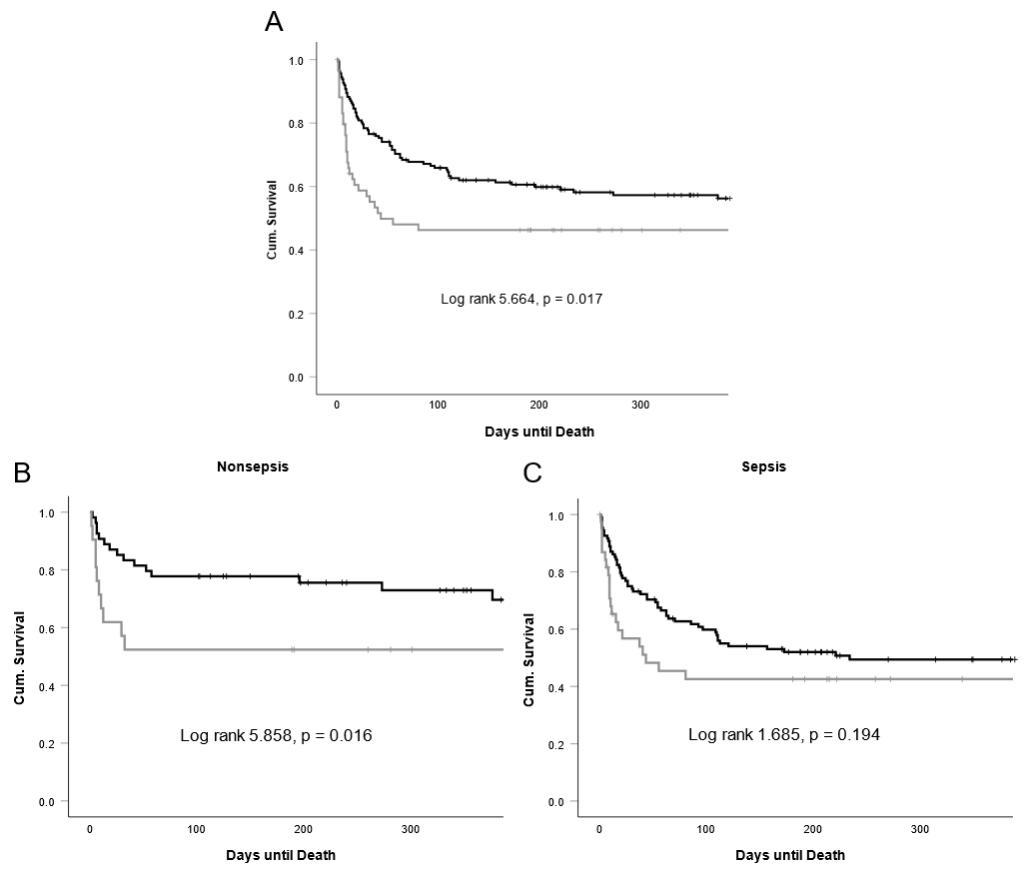
Kaplan–Meier curves for survival of all (**A**), nonseptic (**B**), and septic (**C**) ICU patients depending on levels of SFRP5 < 54.5 ng/mL (black) and ≥ 54.5 ng/mL (grey). Censored events are indicated by a crossing vertical line. Cutoff values of the Kaplan–Meier curve were determined by the Youden index for all ICU patients. Sample sizes: all patients n = 223, nonsepsis n = 76, sepsis n = 147.

We next aimed at understanding whether downregulation or upregulation of circulating SFRP5 could point to different outcomes of critical care patients. Hence, we analyzed SFRP5 serum concentrations at admission with respect to survival at consecutive standardized time points (i.e., 30, 60, 90, 180, and 365 days). Here, we could observe an overall trend towards lower levels of SFRP5 in patients that survived the respective timepoint. Only for the timepoint 60 days after admission to the ICU, this reached statistical significance (Figure 4). To further analyze the influence of SFRP5 on survival, we conducted a Kaplan–Meier curve analysis using the Youden index for the calculation of an ideal cutoff value of 54.5 ng/mL. Here, critical care patients with SFRP5 serum concentrations of lower than 54.5 ng/mL showed a more favorable outcome than those with levels higher or equal to 54.5 ng/mL (log rank 5.664, *p* = 0.017; Figure 5A). In line with the preceding analysis, curve separation was largest in the beginning and decreased towards the end of the curve (Breslow 8.594, *p* = 0.003; Tarone-Ware 7.077, *p* = 0.008). To further dissect the impact of SFRP5 levels on survival, we also conducted Kaplan–Meier analyses for our study cohorts of septic and nonseptic patients, with the same SFRP5 cutoff value of 54.5 ng/mL. In nonseptic patients, curve separation was retained (log rank 5.858, *p* = 0.016; Breslow 6.067, *p* = 0.014; Tarone-Ware 5.848, *p* = 0.016; Figure 5B). In contrast, we did not observe a clear curve separation in septic patients, except for the time points of approximately 30 to 90 days (Log rank 1.685, *p* = 0.194; Breslow 4.120, *p* = 0.042; Tarone-Ware 2.981, *p* = 0.084; Figure 5C).

To depict possible associations to other adipokines we analyzed five other adipokines in a subset of 91 patients. Here, we observed a moderate negative correlation between SFRP5 and resistin serum concentrations (Spearman’s r = −0.288, *p* = 0.006, Table 5). Other adipokines, namely adiponectin, ghrelin, leptin, and the leptin receptor did not correlate with SFRP5 serum concentrations in this subset of patients (Table 5).

## 4. Discussion

Dysregulation of the immune system plays a crucial role in the development of sepsis [9]. Unfortunately, despite pioneering research, those pathophysiological mechanisms of immune regulation are not sufficiently understood, and this remains an unmet need for improved prognostication and therapy of this deadly disorder [41]. The dysregulation of the Wnt5a / SFRP5 system has already been demonstrated in a smaller study of 60 critically ill patients with sepsis [37]. However, alterations of SFRP5 concentrations have not been shown. In this study, we present lower serum levels of the anti-inflammatory adipokine SFRP5 in critically ill and septic patients for the first time, reflecting the initial pro-inflammatory state. This was even more pronounced in sepsis, as septic patients showed continuously lower SFRP5 levels than nonseptic patients. Furthermore, septic patients recovered levels of SFRP5 in the time course of sepsis. Regarding survival, nonseptic patients with lower levels of SFRP5 had higher survival rates as compared to those with higher levels. Interestingly, SFRP5 did not show any relevant prognostic character in sepsis in our study validating the results of the previous study [37]. SFRP5 levels also correlated to the length of ICU stay, leukocytes, anti-inflammatory markers, as well as fat and protein metabolism.

Regulation of the immune systems exhibits a key role in the prognosis of critical illness and sepsis [9]. In line with previous findings [37], we were able to show reproduction of the described dysregulation of the Wnt5a/SFRP5 system. Likewise, previous studies have reported negative correlations of SFRP5 to obesity, HbA1c and HOMA-IR [10]. In our ICU cohort, we did not find correlations between those parameters or diabetes as reported before [34], suggesting that SFRP5 is influenced by other overlaying factors in acute disease. However, lower concentrations of SFRP5 have been demonstrated in coronary artery disease (CAD) [33] as well as higher levels of SFRP5 in chronic heart failure, measured by left ventricular end diastolic diameter (LVEDD) or levels of NT-proBNP [36]. In this study, patients with CAD also showed a tendency of lower levels of SFRP5, although this did not reach statistical significance. Moreover, in our critically ill cohort, NT-proBNP levels did not correlate with SFRP5 serum concentrations. Unfortunately, measurements of LVEDD were not available for analysis. Previous data additionally suggest regulation of the Wnt5a/SFRP5 system in liver disease including non-alcoholic fatty liver disease (NAFLD) [10]. We now demonstrate higher levels of SFRP5 in critically ill patients with liver disease. Furthermore, older patients also exhibited lower levels of SFRP5. In addition to those findings, we enhanced the knowledge and understanding of the Wnt5a/SFRP5 system through our study of a few key findings. Firstly, the Wnt5a/SFRP5 system is not only dysregulated in sepsis alone, but also in critical illness in general. We enlarged current knowledge of the Wnt5a/SFRP5 system by demonstrating lower levels of SFRP5 in patients with critical illness. Secondly, SFRP5 is downregulated the strongest in septic patients. This fully supports the idea of multifactorial anti-inflammatory regulation in early sepsis. Thirdly, septic patients seem to restore levels of SFRP5 during sepsis, reflecting the later stages of anti-inflammation. Lastly, SFRP5 concentrations below 54.5 ng/mL at admission seem to be beneficial for the outcome of critically ill patients. Notably, patients with sepsis did not show significantly better prognosis with lower levels of SFRP5, as opposed to nonseptic critically ill patients, who showed far better outcomes in this situation. This striking result points towards dysregulation of the immune system not only in sepsis, but in critical illness in general. In nonseptic patients, initial anti-inflammation seems to be disadvantageous regarding survival.

It is important to openly discuss the limitations of our study. We conducted a single-center study with a limited number of patients. This led to high reproducibility in measurements but restricted the possibility of more in-depth subgroup analyses. However, we could not reproduce all previously described correlations of SFRP5 to chronic diseases e.g., diabetes or chronic heart failure, in the intensive care setting [34,36]. The upper cut-off of SFRP5 for the assay used was set at 100 ng/mL. Many measurements were situated right at the cut-off value and therefore even well above. Measuring SFRP5 levels higher than 100 ng/mL would most likely enhance understanding of the distribution of concentrations. We observed several correlations to other biomarkers (e.g., inflammation and metabolism) which showed medium strength at most. Here, the clinical value must be carefully evaluated. Future and larger multi-center studies of this biomarker should be conducted, aiming at a deeper understanding of the role of SFRP5 in different subgroups of critically ill patients.

## 5. Conclusions

Our study demonstrated decreased SFRP5 serum concentrations in a well described cohort of critically ill patients at admission to the ICU as compared to healthy individuals. Moreover, we observed even lower levels of serum SFRP5 in septic patients as compared to nonseptic critically ill patients. SFRP5 is associated with metabolic and inflammatory alterations and is an indicator for mortality but not disease severity. Interestingly, we found the strongest influence on mortality in nonseptic patients, possibly underlining the role of anti-inflammatory processes in nonseptic critically ill patients.

Potential future investigations could focus on describing the role of anti-inflammatory processes such as the adipokine SFRP5, not only in septic, but also in nonseptic critically ill patients.

## Figures and Tables

**Table 1:** Baseline patient characteristics.

Parameter	All Patients	Sepsis	Nonsepsis	*p*-Value
Number n	223	147	76	
Sex (male/female) n	134/89	87/60	47/29	0.701
Age (years)	63 (18–90)	64 (20–90)	61 (18–85)	0.587
APACHE II score	18 (3–43)	19 (3–43)	15 (3–33)	0.003 *
SOFA score	10 (0–19)	11 (3–19)	8.5 (0–16)	0.008 *
Charlson Comorbidity Index	5 (0–12)	5 (0–12)	5 (0–11)	0.433
Mechanical ventilation n (%)	166 (74.4)	111 (75.5)	55 (72.4)	0.610
Vasopressor demand n (%)	152 (68.2)	109 (74.1)	43 (56.6)	0.008 *
ICU days n	9 (2–137)	12 (2–137)	7 (2–45)	0.002 *
Death in ICU n (%)	54 (24.2)	38 (25.9)	16 (21.1)	0.428
30-day mortality n (%)	61 (27.4)	43 (29.3)	18 (23.7)	0.374
1-year mortality n (%)	98 (43.9)	74 (50.3)	24 (31.6)	0.015 *
SFRP5 (ng/mL)	24.66 (0.06–100)	19.21 (0.06–100)	32.83 (0.10–100)	0.031 *

The median and range (in parentheses) are given, unless indicated otherwise. Abbreviations: APACHE: acute physiology and chronic health evaluation; SOFA: sequential organ failure assessment; ICU: intensive care unit; SFRP5: secreted frizzled related protein 5; * Significance between sepsis and nonsepsis patients was assessed using the Mann-Whitney U test or chi-squared test, respectively. *p*-values < 0.05 were considered statistically significant and were highlighted (“*”).

**Table 2:** Disease etiology of the study population.

Etiology of (non)Sepsis Critical Illness, n (%)	Sepsis n = 147	Nonsepsis n = 76
Pulmonary	83 (56.5)	
Abdominal	22 (15.0)	
Urogenital	10 (6.8)	
Other	32 (21.8)	
Cardiocirculatory disorder		17 (22.4)
Respiratory failure		12 (15.8)
Advanced liver disease		17 (22.4)
Other		30 (39.5)

The absolute numbers and percentage of the respective subgroup (in parentheses) are given.

**Table 3:** Correlations of clinical and laboratory parameters with SFRP5 serum concentrations at ICU admission.

Parameters	r	*p*-Value
**Demographics**		
Age	−0.138	0.040 *
Body mass index	0.022	0.755
**Blood count and markers of inflammation**		
Leukocytes	0.133	0.048 *
Hemoglobin	0.140	0.036 *
Platelets	0.034	0.616
C-reactive Protein	−0.088	0.190
Procalcitonin	−0.175	0.028 *
Interleukin 6	−0.061	0.435
Interleukin 10	−0.302	<0.001 *
**Electrolytes and renal system**		
Sodium	0.083	0.219
Potassium	0.084	0.212
Urea	0.047	0.489
Uric acid	0.053	0.480
Creatinine	−0.004	0.953
Cystatin C	0.021	0.772
**Hepato-pancreatico-biliary system and coagulation**		
Protein, total	0.238	<0.001 *
Albumin	0.228	0.008 *
INR	−0.030	0.662
aPTT	−0.154	0.023 *
Bilirubin, total	0.110	0.113
Gamma-GT	0.093	0.166
AST	0.095	0.172
ALT	0.117	0.084
Lipase	0.147	0.049 *
**Cardiopulmonary system**		
NTproBNP	−0.003	0.978
Norephinephrine demand at day 1 (µg/day)	−0.017	0.816
Horovitz quotient (PaO_2_/FiO_2_)	0.049	0.724
Ventilatory FiO_2_ demand	0.156	0.161
Net fluid balance day 1	−0.059	0.406
Net fluid balance day 3	−0.070	0.332
**Metabolism and endocrinology**		
Glucose	−0.039	0.564
HbA1c	0.116	0.242
Insulin	0.123	0.219
C-Peptide	0.004	0.966
HOMA IR	0.021	0.838
Cholesterol	0.173	0.019 *
HDL-cholesterol	0.201	0.046 *
LDL-cholesterol	0.265	0.008 *
Triglycerides	0.058	0.436
**ICU parameters**		
Days on ICU	−0.155	0.021 *
SOFA day 1	0.027	0.803
SOFA day 3	−0.064	0.585
APACHE-II day 1	−0.034	0.661
APACHE-II day 3	−0.097	0.391

Spearman rank correlation test was used to calculate significant correlations of positive and negative nature. *p*-values < 0.05 were considered statistically significant and were highlighted (“*”). Abbreviations: ICU: intensive care unit; INR: International normalized ratio; aPTT: activated partial thromboplastin time; Gamma-GT: Gamma-glutamyl transpeptidase; AST: Aspartate aminotransferase; ALT Alanine aminotransferase; NTproBNP: N-terminal pro B-type natriuretic peptide; PaO2: arterial partial pressure of oxygen; FiO2: Fraction of inspired oxygen; HbA1c: Glycosylated hemoglobin A1; HOMA-IR: Homeostatic model assessment–insulin resistance; HDL: high-density lipoprotein; LDL: low-density lipoprotein; SOFA: Sepsis-related organ failure assessment; APACHE-II: acute physiology and chronic health evaluation II.

**Table 4:** Comorbidities and their influence on SFRP5 levels.

Comorbidity	SFRP5 Concentration in ng/mL, Median (Range)	*p*-Value
Diabetes	22.13 (0.06–100)	0.972
Liver disease	47.77 (6.59–100)	0.005 *
Coronary artery disease	21.54 (0.06–100)	0.063
Hypertension	25.44 (0.10–100)	0.729
Chronic alcohol abuse	34.68 (3.28–100)	0.077
Chronic obstructive lung disease	17.44 (1.94–100)	0.497
Active malignancy	13.09 (0.06–100)	0.056

The median and range (in parentheses) are given, unless indicated otherwise. * Significance between groups was assessed using the Mann–Whitney U test. *p*-values < 0.05 were considered statistically significant and were highlighted (“*”).

**Table 5:** Correlations of SFRP5 serum concentrations with adipokines at ICU admission.

Adipokine	r	*p*-Value	n
Adiponectin	0.169	0.109	91
Ghrelin	0.101	0.350	88
Resistin	−0.288	0.006 *	91
Leptin	0.069	0.517	91
Leptin receptor	0.178	0.093	90

Spearman rank correlation test was used to calculate correlations of positive and negative nature. *p*-values < 0.05 were considered statistically significant and were highlighted (* indicates *p* < 0.05).

## Data Availability

The data presented in this study are available on request from the corresponding author.
